# A new method of infrared thermography for quantification of brown adipose tissue activation in healthy adults (TACTICAL): a randomized trial

**DOI:** 10.1007/s12576-016-0472-1

**Published:** 2016-07-21

**Authors:** Qi Yan Ang, Hui Jen Goh, Yanpeng Cao, Yiqun Li, Siew-Pang Chan, Judith L. Swain, Christiani Jeyakumar Henry, Melvin Khee-Shing Leow

**Affiliations:** 1 Clinical Nutrition Research Centre, Singapore Institute for Clinical Sciences, Agency for Science, Technology and Research (A*STAR), Singapore, Republic of Singapore; 2 Institute for Infocomm Research, Agency for Science, Technology and Research (A*STAR), Singapore, Republic of Singapore; 3 Department of Medicine, Yong Loo Lin School of Medicine, National University of Singapore, Singapore, Republic of Singapore; 4 School of Engineering and Mathematical Sciences, La Trobe University, Melbourne, Australia; 5 Department of Biochemistry, Yong Loo Lin School of Medicine, National University of Singapore, Singapore, Republic of Singapore; 6 Department of Endocrinology, Tan Tock Seng Hospital, Singapore, Singapore; 7 Lee Kong Chian School of Medicine, Nanyang Technological University, Singapore, Republic of Singapore; 8 Office of Clinical Sciences, Duke–NUS Graduate Medical School, Singapore, Republic of Singapore

**Keywords:** Brown adipose tissue, Supraclavicular skin temperature, Infrared thermography, Capsinoids, Cold challenge

## Abstract

The ability to alter the amount and activity of brown adipose tissue (BAT) in human adults is a potential strategy to manage obesity and related metabolic disorders associated with food, drug, and environmental stimuli with BAT activating/recruiting capacity. Infrared thermography (IRT) provides a non-invasive and inexpensive alternative to the current methods (e.g. ^18^F-FDG PET) used to assess BAT. We have quantified BAT activation in the cervical-supraclavicular (C-SCV) region using IRT video imaging and a novel image computational algorithm by studying C-SCV heat production in healthy young men after cold stimulation and the ingestion of capsinoids in a prospective double-blind placebo-controlled randomized trial. Subjects were divided into low-BAT and high-BAT groups based on changes in IR emissions in the C-SCV region induced by cold. The high-BAT group showed significant increases in energy expenditure, fat oxidation, and heat output in the C-SCV region post-capsinoid ingestion compared to post-placebo ingestion, but the low-BAT group did not. Based on these results, we conclude that IRT is a promising tool for quantifying BAT activity.

**Electronic supplementary material:**

The online version of this article (doi:10.1007/s12576-016-0472-1) contains supplementary material, which is available to authorized users.

## Introduction

White adipose tissue (WAT) is the primary site of energy storage in humans, while the function of brown adipose tissue (BAT) is specifically to transfer energy from food into heat, i.e. thermogenesis [1]. BAT refers to both classical brown adipocytes and ‘beige’ adipocytes (also termed ‘brite’ adipocytes or inducible brown adipocytes) [2]. Human BAT depots contain both brown-like and beige-like adipocytes [3, 4], both of which we refer to as BAT in this study.

BAT dissipates energy as heat (non-shivering thermogenesis) by uncoupling oxidative phosphorylation, a process that is often stimulated by exposure to cold [5–7]. In humans, BAT is especially prevalent in newborn babies, functioning as a defense against hypothermia, but its prevalence diminishes greatly with age. Early researchers believed that the abundance of BAT was so low as to be negligible in adult humans, but more recent studies demonstrate the existence of BAT in healthy adults [8–11], with the cervical–supraclavicular (C-SCV) region identified as the largest and most metabolically active BAT depot in adult humans.

There is much interest in discovering foods, drugs, and environmental stimuli that can recruit and activate BAT as a therapeutic strategy against obesity and obesity-linked diseases. It is well documented that capsaicin and capsinoids, all substances naturally present in chili pepper, stimulate thermogenesis and/or fat oxidation [12, 13] via stimulation of the transient receptor potential vanilloid subtype 1 [14], resulting in increased sympathetic efferent activity and thereby BAT thermogenesis [15, 16]. Capsinoids have also been reported to upregulate uncoupling protein 1, a key transmembrane molecule for BAT thermogenesis [16]. Taken together, it would appear that capsinoids increase energy expenditure through activation of BAT [17].

The current ‘gold standard’ available to assess BAT activity is ^18^F-fluorodeoxyglucose positron emission tomography-computed tomography (FDG PET/CT) fusion imaging, which uses glucose uptake as a proxy for BAT activity. However, PET/CT imaging has several well-recognized limitations and provides an incomplete picture of BAT metabolic activity [18]. PET/CT studies require costly equipment that is often primarily used for clinical care and only secondarily available for research studies; in addition, such equipment is often located at a distance from dedicated human research units. These studies are also costly to perform, and thus this strategy is not feasible for large-scale screening studies. The radiation exposure with PET limits its use, especially among healthy people; consequently, rather than performing pre- and post-intervention studies in individual subjects, often only one PET scan is performed in an individual who has either been assigned to a control or intervention group. In addition, serial PET scans to assess the kinetics of rapid BAT activation and deactivation cannot be performed.

Given that the main function of BAT is thermoregulation (with heat production as the final downstream end-product), measuring changes in BAT temperature is the fundamental basis of quantifying BAT activation. In rodents, such changes can be measured directly by placing thermistors directly into the BAT depots [19]; however, this is an invasive method and is not suitable for human studies. An attractive alternative method is to measure the temperature of the skin overlaying BAT depots, based on heat transfer from underlying BAT towards the skin [20]. Studies employing infrared thermography (IRT) as a non-invasive method of assessing human BAT activity are emerging [21–23], and the utility of IRT in detecting human BAT has been validated against PET/CT imaging in a recent study [23]. However, IRT for BAT assessment is a nascent field without established IR imaging protocols or image processing methods, with most studies using the average supraclavicular skin temperature estimated from IR still pictures which, unfortunately, yield an incomplete surrogate reflection of heat production by BAT.

In the study reported here, we applied a novel method of quantifying BAT heat production using IRT video imaging, coupled with an algorithm for determining the volume and degree of activation of BAT. We used this IRT method to investigate skin temperature changes and consequently BAT heat production within the C-SCV region in human adults under the conditions of cold stimulation and consumption of capsinoids, respectively.

## Methods and subjects

### IR video processing and thermal energy quantification

Based on BAT thermogenesis causing in an increase in skin temperature, a number of previous studies used a threshold segmentation technique to detect regions of increased IR signal, with the threshold calculated as the median value of the highest 25 % of pixels [22, 24]. However, this approach neglects spatial information in the image and cannot generate accurate data in terms of the detection results for analysis of BAT activity. In our study we modified the seeded region growing (SRG) technique [25] and applied it to IR video imaging to detect local regions of interest (ROIs), a term we use to refer to the ‘hot’ regions overlaying the C-SCV BAT depots that are delineated by our algorithm.

**Fig. 1:**
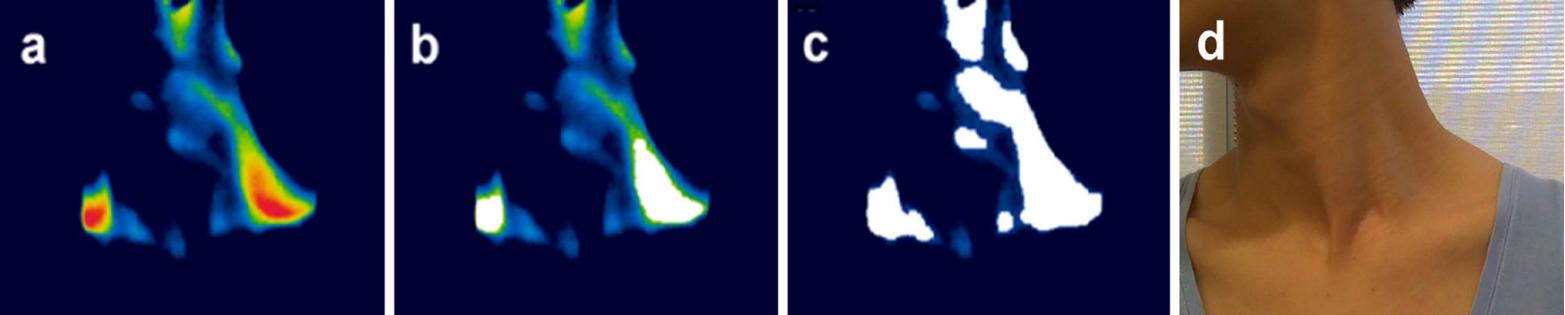
Comparison of methods to detect the regions of interest (ROIs) of brown adipose tissue (BAT). **a** An infrared (IR) thermal image of BAT over the cervical–supraclavicular (C-SCV) region, **b** the BAT ROI as determined applying the modified seeded region growing (SRG) algorithm in the current study, **c** BAT ROI determined using the threshold segmentation technique, **d** a digital image of the same individual. Color figure online

The standard SRG algorithm starts with the selection of a suitable seed and further expands it into spatially connected regions of similar characteristics. In our application, the seed is automatically determined by searching for the pixel of the highest temperature in the pre-defined image areas (e.g., the left or right C-SCV region). To increase the robustness of the SRG algorithm, we define a small local region $A_{I}$ containing the seed pixel to ensure that a stable mean temperature can be estimated for the region. Let *H* be the set of all unallocated pixels that are adjacent to region *A*_*I*_:

1 $$H = \left\{ {\varvec{x} \notin A_{i} |N\left( \varvec{x} \right) \cap A_{i} \ne \emptyset } \right\},$$

where $N\left( \varvec{x} \right)$ is the 8-neighbors of the pixel $\varvec{x}$. For a pixel $\varvec{x} \in H$, the SRG algorithm computes the difference $\delta \left( \varvec{x} \right)$ between its temperature value and the mean temperature of the region $A_{i }$ as a measure of how different pixel $\varvec{x}$ is from the region it adjoins:

2 $$\delta \left( \varvec{x} \right) = \left| {T\left( \varvec{x} \right) - \mathop {\text{mean}}\limits_{{\varvec{y} \in A_{i} }} \left[ {T\left( \varvec{y} \right)} \right]} \right|,$$

where $T\left( \varvec{x} \right)$ is the temperature reading of pixel $\varvec{x}$. We then take $\varvec{z} \in H$ such that

3 $$\delta \left( \varvec{z} \right) = \mathop {\hbox{min} }\limits_{{\varvec{x} \in H}} \left[ {\delta \left( \varvec{x} \right)} \right]\,\quad{\text{ and}}\quad\delta \left( \varvec{z} \right) \le T_{t} ,$$

and add pixel $\varvec{z }$ into the region *A*_*i*_. This iterative process is repeated until $\delta \left( \varvec{z} \right)$ becomes larger than a pre-defined threshold $T_{t}$. Different from the standard application of SRG which involves assigning all unallocated pixels to the most homogenous regions, our application requires the detection of local ROIs which contain spatially connected pixels of similar temperature values. Therefore, we set the parameter $T_{t }$ to control how tolerant the SRG algorithm will accept a neighboring pixel to grow the region. In our study, $T_{t }$ was optimized for individual subjects to achieve reliable segmentation. In Fig. 1, we show the results for ROI detection and compare these with results based on the threshold segmentation technique. We demonstrate that our method can accurately detect local pixel clusters with closed boundaries overlaying the BAT depot in the C-SCV region. In comparison, the threshold segmentation technique can only identify a number of disconnected pixel clusters, a limitation which reduces its reproducibility and accuracy.

We applied this algorithm to 5 s of IR video sequence per data point, with a total of 150 frames per video sequence. Only 5 s of IR video sequence per data point was used in this pilot study since processing IR video sequences was a time-consuming process, with longer video sequences requiring longer processing times. Our algorithm calculated the number of pixels and the mean temperature value of pixels in the ROIs detected in each frame and then averaged these values across the entire 5 s (150 frames) of video sequence. The pixel count of the ROI was used to estimate the actual area of the ‘hot region’ overlaying the C-SCV BAT on the human body based on a simple calibration. Two 5-mm aluminum foil disks placed on the skin—one under each clavicle symmetrically spaced 18 cm apart and two other similar disks each positioned 8 cm above the respective subclavicular disks on the superior border of the trapezius muscle bilaterally—served as fiducial markers delineating a rectangle on a three-dimensional plane. A 3×3 matrix could then be computed to represent transformation between two spatial frames of reference for the registration of images against known linear dimensions and enable subsequent computation of the ROI area via pixel counts. As the aluminum foil disks had a distinct temperature of ~28–31 °C, compared to skin temperatures image registration with these fiducial markers also permitted precise detection of changes in the actual temperature to be distinguished from those caused by changes in body poses.

We next quantified BAT heat energy output in watts (W) by applying the Stefan–Boltzmann law:

4 $${\text{BAT heat energy output }}\left( {\text{W}} \right) \, = \varepsilon \times \sigma \times \, A \, \times \, T^{4} ,$$

*ε*: emissivity (0.98 for human skin);

σ: Stefan–Boltzmann’s constant (5.676 × 10^−8^ W/m^2^K^4^);

A: mean ROI area in m^2^ (based on number of pixels in detected ROI);

T: mean ROI temperature in Kelvin (averaged temperature values of all pixels in detected ROI).

The heat energy output of the left and right C-SCV BAT were summed to give the total heat energy emitted by the C-SCV BAT depots.

### IRT of C-SCV regions

Subjects were seated in a relaxed and upright posture with arms adducted, away from all heat-emitting objects and at a 1.0-m distance from an IR thermal imaging camera fastened onto a tripod (model FLIR T440; FLIR Systems AB, Täby, Sweden) with thermal resolution at 76,800 (320 × 240) pixels. This positioning was to assure optimum visualization of the C-SCV regions. Thermal imaging was performed in the same room as the indirect calorimetric measurements, at a constant ambient temperature of 24 °C.

All IR thermal video recordings were performed over a standard recording period of 5 min. During the 5-min recording period, subjects were asked on three separate occasions to turn their heads to the right for 10 s while keeping shoulders still in the antero-posterior orientation, then instructed to turn their heads to their left for another 10 s in a similar fashion before turning their heads back to face the camera. This sequence of changes in head position was to allow for imaging of the front and both antero-lateral views of the C-SCV regions.

On the first visit, thermal imaging of the C-SCV regions was carried out while the subjects were exposed to a cold stimulus for 5 min. The cold stimulation consisted of the immersion of both hands and feet in water kept at 18 °C, a local cooling protocol adapted from Symonds et al. [22].

Capsinoid ingestion trials were conducted on each of the two subsequent test visits. Thermal data from the C-SCV regions were recorded continuously for 5 min before each subject ingested the test meal to obtain the baseline temperature. IRT (5 min per recording) was repeated at 30-min intervals over a 2.5-h period following ingestion of capsinoids or placebo. All thermal data were recorded in a radiometric IR video format and analyzed using the FLIR research IR software [26]. The IR videos were recorded at 30 frames per second.

### Subjects

Between October 2013 and June 2014, 24 healthy, lean [body mass index (BMI) of <25 kg/m^2^] male volunteers were recruited (Table 1). Potential subjects underwent a screening session consisting of a questionnaire and measurement of BMI and fasting blood glucose. Exclusion criteria included pre-diabetes or diabetes, metabolic disorders, ingestion of medications, smoking, and recent weight change. This trial was registered at http://www.clinicaltrials.gov (ID: NCT01961674; registration date: 10/10/2013).

**Table 1:** Baseline characteristics of subjects

Characteristics	All (*n* = 24)	Low-BAT group (*n* = 18)^a^	High-BAT group (*n* = 6)^a^
Age (year)	23 (±0.4)	23 (±0.4)	22 (±0.5)
Body weight (kg)	59 (±1.4)	60 (±1.7)	57 (±1.7)
Height (m)	1.70 (±0.14)	1.70 (±0.17)	1.71 (±0.10)
BMI (kg/m^2^)	20.4 (±0.3)	20.6 (±0.3)	19.7 (±0.6)
Percentage body fat (BODPOD®)^b^	14.4 (±1.0)	15.0 (±1.2)	12.5 (±1.2)
Fasting blood glucose (mmol/L)	4.4 (±0.1)	4.4 (±0.1)	4.2 (±0.1)
Resting EE before capsinoids ingestion (kcal/day)	1483 (±36)	1505 (±47)	1410 (±70)
Resting EE before placebo ingestion (kcal/day)	1478 (±39)	1504 (±43)	1430 (±65)

All values are presented as the mean (± standard error)

*BAT* Brown adipose tissue,* BMI* body mass index, *EE* energy expenditure

^a^Subjects were exposed to 5 min of cold stimulation by immersion of both hands and feet in 18 °C water and the cervical–supraclavicular (C-SCV) regions imaged (infrared thermography). The increase in heat production was calculated and compared to the baseline values. Subjects were then divided into two groups: those who showed a relatively smaller percentage change in heat production ( (low-BAT group) and those who showed a greater percentage increase in heat production (high-BAT group). Subjects with a percentage change in heat production of more than the average +1 standard error (SE) were arbitrarily categorized into the high-BAT group. Student’s *t* test showed no significant differences in other variables between the low-BAT and high-BAT groups

^b^BOD POD® Body composition measurement test (COSMED, Rome, Italy)

### Test substances

Capsinoids and placebo capsules were provided by Ajinomoto Inc. (Tokyo, Japan). The capsinoids were extracted from the fruit of sweet chili pepper *Capsicum anuum L*. var. CH-19 plants, purified, and encapsulated as previously described [27]. Each gel capsule contained either placebo or 1.5 mg of capsinoids; the latter capsules contained capsiate, dihydrocapsiate, and nordihydrocapsiate in a 7:2:1 ratio, and 199 mg of a mixture of rapeseed oil and medium-chain triglycerides.

### Study protocol

This was a double-blinded, placebo-controlled, randomized crossover study in which each subject completed two study visits and consumed (in random order) either 0 or 9 mg of capsinoids in capsule form. All capsules looked identical and contained either 0 (placebo) or 1.5 mg of capsinoids. Six capsules (1.5 mg each) were consumed during each test visit, together with a standardized portion of white rice containing 50 g carbohydrates. The ingestion of rice enabled a comparison of the thermic effect of food with diet-induced thermogenesis triggered by capsinoid-stimulated BAT as well as an assessment of whether BAT activation had any effect on glycemic response. Randomization was performed using a computer-generated random number list prepared by one of the authors with no clinical involvement in the study, such that both subjects and investigators involved in the study were blinded to the capsule assignment for each study visit.

On each test visit, subjects reported to the laboratory between 0800 and 0900 hours after an overnight fast of at least 10 h. Fasting blood glucose was measured at each visit using a glucose dehydrogenase method (HemoCue AB, Angelholm, Sweden). The mean fasting blood glucose level was determined by averaging the fasting blood glucose values obtained on both test visits. Subjects were asked to avoid strenuous physical activity, alcohol consumption, and consumption of chili or spicy foods on the day prior to the test visits.

**Fig. 2:**
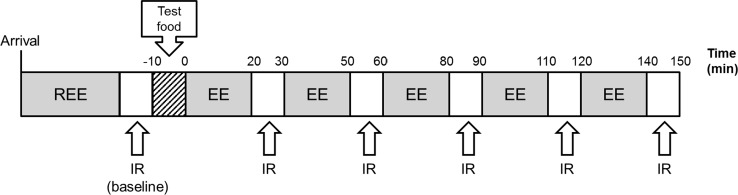
Schematic of study visit protocol. During the study visit, the resting energy expenditure (*REE*) of each subject (after an overnight fast) was first measured by indirect calorimetry, and the baseline heat production by BAT was measured based on IR thermography imaging of the C-SCV region. Subjects then consumed a portion of white rice together with capsules containing either placebo or capsinoids (9 mg). The EE was monitored over the next 2.5 h, with IRT imaging performed at half-hourly intervals

Upon arriving at the laboratory, subjects were provided standard cotton singlets (to ensure adequate exposure of the neck and upper thorax for thermal imaging) and Bermuda shorts with an estimated Clo value of 0.2 [28]. The Clo unit provides a measure of thermal insulation provided by clothing [29]. Room temperature was maintained at 24 °C so that subjects were in thermal comfort conditions to avoid shivering that may confound the results. With the subjects resting in a supine position, oxygen consumption and carbon dioxide production were continuously recorded via indirect calorimetry for 45 min. The stable value of the last 10-min period was used to calculate the respiratory quotient (RQ) and resting energy expenditure (EE), the latter considered to be a good approximation of the basal metabolic rate (BMR). After 45 min, IR imaging of the C-SCV regions was performed by video recording for 5 min. The subjects then ingested capsules containing the placebo or 9 mg of capsinoids, with water ad libitum and a portion of white rice. During the 2.5 h following the consumption of the capsules and food, respiratory gas parameters were recorded continuously for 20 min of each of five 30-min time intervals, and the EE and RQ were recorded during the last 10-min period of each 30-min time interval. At 30-min intervals, IR imaging of the C-SCV regions was performed over a standard recording period of 5 min (see Fig. 2).

### Indirect calorimetry

Indirect calorimetry (Quark RMR; COSMED) was used to provide a sensitive imaging-independent proxy of BAT activation. Changes in EE and RQ were measured since UCP-1-dependent heat production should elevate the metabolic rate and thus increase EE. Oxygen consumption and carbon dioxide production were measured using a ventilated hood system, with the subjects resting in a supine position. EE and RQ were estimated from measures of oxygen consumption and carbon dioxide production based on the Weir equation [30].

After an overnight fast, resting EE was measured for 45 min prior to consumption of the test meal (6 capsules + standardized portion of white rice). Following the test meal, postprandial EE was measured in 20-min blocks over the next 2.5 h.

### Statistical analysis

Paired *t* tests were used to ascertain if there were differences in SCV heat production under cold stimulation conditions between the low-BAT (placebo) and high-BAT (capsinoids) groups. Data were expressed as mean ± standard error of the mean (SEM). A multi-level model constructed with the generalized structural equation model (gSEM) framework [31] was used to determine if there were significant differences in EE, fat oxidation, and SCV heat production between the capsinoids and placebo groups over time (0.5, 1, 1.5, 2, 2.5 h) in low-BAT, high-BAT, and all subjects, while adjusting for the respective baseline values. The coefficients were interpreted as the average difference in these outcomes between the capsinoids and placebo (reference) groups. The standard errors of the coefficients were adjusted with a robust procedure. As an advanced regression model, gSEM is ideal for handling longitudinal data where the outcomes are monitored over time as it captures all salient features (treatment effects, time effects, baseline effects), thus better reflecting the nature of our data [32]. Notably, gSEM has a higher statistical power and is superior to random-effects, repeated-measures analysis of variance since it captures more features and requires fewer assumptions, which therefore optimizes its capability of detecting a statistically significant difference even with a small sample size. All analyses were performed with Stata/MP version 14 (Stata Corp, College Station, TX), and the significance level for all statistical tests was set at 5 %.

## Results

### IRT quantification of cold-stimulated heat production in the C-SCV region

**Fig. 3:**
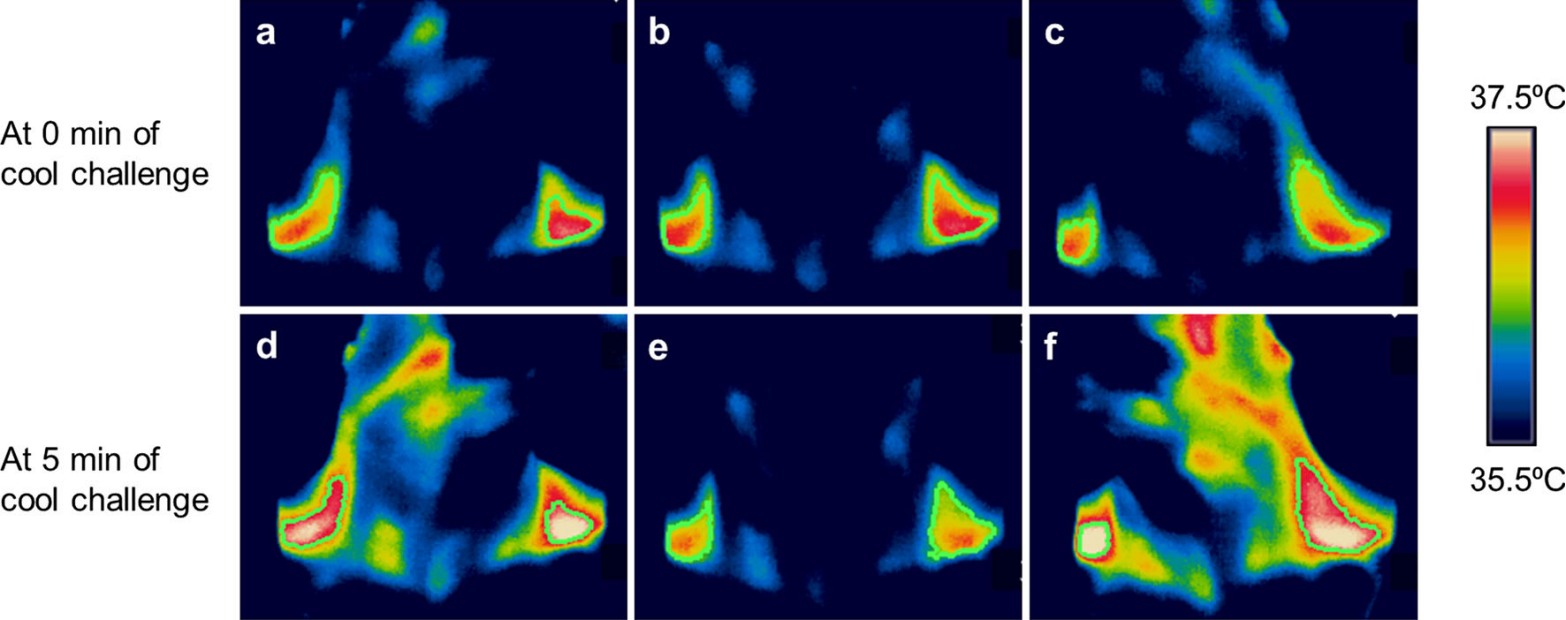
Thermal images of cold-induced BAT activity. Representative images taken at 0 min (**a**, **b**, **c**) and 5 min (**d**, **e**, **f**) of cold challenge, showing an increase in the area and temperature of the thermally active region. The subject’s head was turned to his left in **a** and **d**, was facing forward in **b** and **e**, and turned to his right in **c** and **f**.* Green contour lines* circumscribe the ROIs over the C-SCV regions, *white pixels* >37.4 °C, *red pixels* 36.8–37.4 °C; *yellow pixels*, 36.5–36.8 °C. Color figure online

Twenty-four subjects were exposed to cold stimulation by immersion of both hands and feet in water maintained at 18 °C, and during this time their C-SCV regions were imaged using IRT. In most subjects, we observed an increase in IRT-measured temperatures over the C-SCV regions at the end of the 5-min cooling protocol (Fig. 3). Higher average temperatures were detected in the ROIs in anterolateral views (Fig. 3a, c, d, f) of the C-SCV regions compared to those in front views (Fig. 3b, e), suggesting that imaging the anterolateral views of the C-SCV could capture more of the heat emanating from BAT. We therefore used thermal data from the anterolateral views of the C-SCV regions (both right and left) for quantification of heat production.

**Fig. 4:**
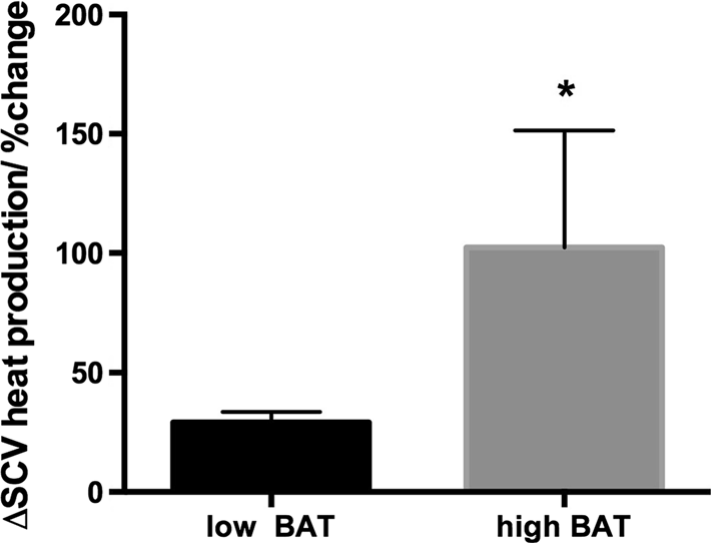
Effect of cold challenge on heat production in the C-SCV region in low-BAT and high-BAT groups (see footnote to Table 1 for definition of groups). *Asterisk* indicates significant difference from low-BAT group by Student’s *t* test at *p* < 0.05. Values are shown as the mean ± standard error (SE)

At the end of the 5-min cooling protocol we calculated the increase in heat production in the C-SCV regions and compared the values to the baseline values. Based on this comparison, we divided the subjects into two groups: those who showed a relatively smaller percentage change in heat production, which was considered to indicate low metabolic activity in the BAT depot (low-BAT group; *n* = 18) and those who showed a greater percentage increase in heat production (high-BAT group; *n* = 6). Subjects with a percentage change in heat production of more than the average +1 SE were arbitrarily categorized into the high-BAT group. Overall, the percentage change in heat production in the C-SCV regions after cold stimulation was significantly greater in the high-BAT group than in the low-BAT group (Fig. 4). The characteristics of the subjects in the low-BAT and high-BAT groups are shown in Table 1, and the changes in ROI temperatures and areas used to calculate the cold-stimulated heat production in the C-SCV regions in the low-BAT and high-BAT groups are shown in Table 2.

**Table 2:** Changes in the temperature, area, and heat energy output of the regions of interest in the left and right cervical–supraclavicular regions in the low-BAT and high-BAT groups during cold challenge

Variables	Low-BAT group	High-BAT group
ΔTemperature (°C)		
Left	0.41 (±0.04)	0.28 (±0.10)
Right	0.43 (±0.04)	0.34 (±0.10)
ΔArea (cm^2^)		
Left	6.29 (±1.68)	11.92 (±1.68)*
Right	6.24 (±1.41)	13.31 (±5.11)
ΔHeat energy output (W)		
Left	0.33 (±0.09)	0.61 (±0.08)*
Right	0.32 (±0.07)	0.68 (±0.26)
ΔTotal heat energy output (W)		
–	0.65 (±0.13)	1.28 (±0.26)*

* Significant difference at *p* < 0.05 (Student’s *t* test) between low-BAT and high-BAT groups

Values are presented as the mean (±SE). Total heat energy output is the sum of the heat energy output from left and right regions of interest (ROIs), calculated based on the Stefan–Boltzmann law. The paired *t* test showed no significant differences between the left and right ROIs

### Effect of capsinoid ingestion on energy expenditure and fat oxidation

The thermogenic effect of capsinoids on EE and fat oxidation was also studied. We noted that the ingestion of capsinoids produced a measurable increase in EE and fat oxidation.

**Fig. 5:**
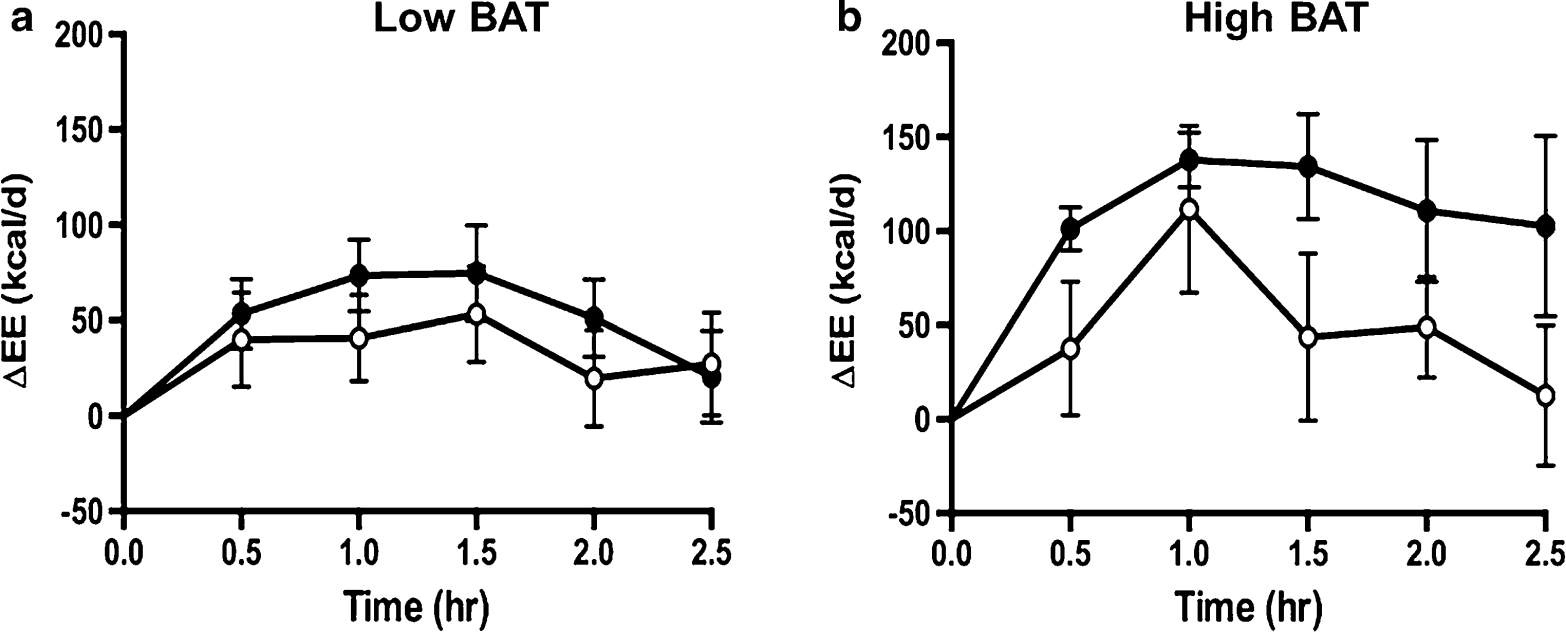
Effect of ingestion of capsules filled with capsinoids on energy expenditure.* Circles* Change in energy expenditure (*ΔEE*) after ingestion of placebo (*open circle*) or 9 mg capsinoids (*filled circle*) together with a standard portion of rice in low-BAT (**a**; *n* = 15) and high-BAT (**b**; *n* = 6) groups. Values are shown as the mean ± SE

 Under the resting condition, prior to the ingestion of capsinoids, the mean EE calculated from oxygen consumption and carbon dioxide production was 1410 ± 70 kcal/day in the high-BAT group; this value did not significantly differ from that in the low-BAT group (1505 ± 47 kcal/day) (Table 1). The change in EE during the 2.5-h period following the ingestion of either capsinoids or placebo, with resting EE taken as the baseline value, in shown in Fig. 5a, b for both the low-BAT and high-BAT groups. Analysis using gSEM showed that in the high-BAT group, the average increase in EE after adjusting for baseline values was significantly higher in subjects who had ingested capsules filled with capsinoids than in those who had ingested capsules filled with placebo over the entire 2.5-h post-ingestion period (coefficient 66.90; *p* = 0.01); this trend was not observed in the low-BAT group (coefficient 26.30; *p* = 0.19) [Electronic Supplementary Material (ESM) Table 1].

**Fig. 6:**
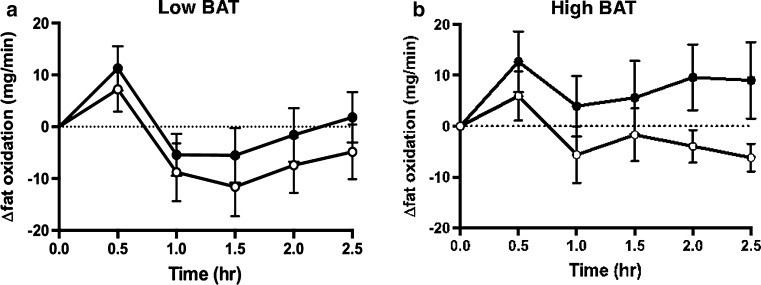
Effect of ingestion of capsinoids on fat oxidation.* Circles* Change in fat oxidation (*Δfat oxidation*) after the ingestion of placebo (*open circle*) or 9 mg capsinoids (*filled circle*) together with a standard portion of rice in the low-BAT (**a**; *n* = 15) and high-BAT (**b**; *n* = 6) groups. Values are shown as the mean ± SE

A similar result was observed for fat oxidation rates calculated from oxygen consumption and carbon dioxide production. Figure 6a, b shows the change in fat oxidation in the low-BAT and high-BAT groups during the 2.5-h period following the ingestion of either capsinoids or placebo. Similar to our findings on EE, the average increase in fat oxidation after adjusting for baseline values was significantly higher after the ingestion of capsinoids compared to placebo only in the high-BAT group (coefficient 11.88; *p* = 0.03)—and not in the low-BAT group (coefficient 5.17; *p* = 0.28) (ESM Table 2).

### Effect of ingestion of capsinoids on IRT-quantified C-SCV heat production

**Fig. 7:**
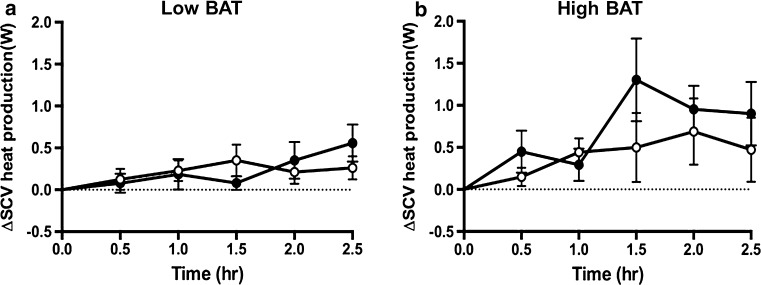
Effect of capsinoids ingestion on heat production in the C-SCV regions as measured by IR thermography. Change in heat energy output in SCV region (*ΔSCV heat production*) after ingestion of placebo (*open circle*) or 9 mg capsinoids (*filled circle*) together with a standard portion of rice in low-BAT (**a**; *n* = 16) and high-BAT (**b**; *n* = 5) groups. Values are shown as the mean ± SE

**Fig. 8:**
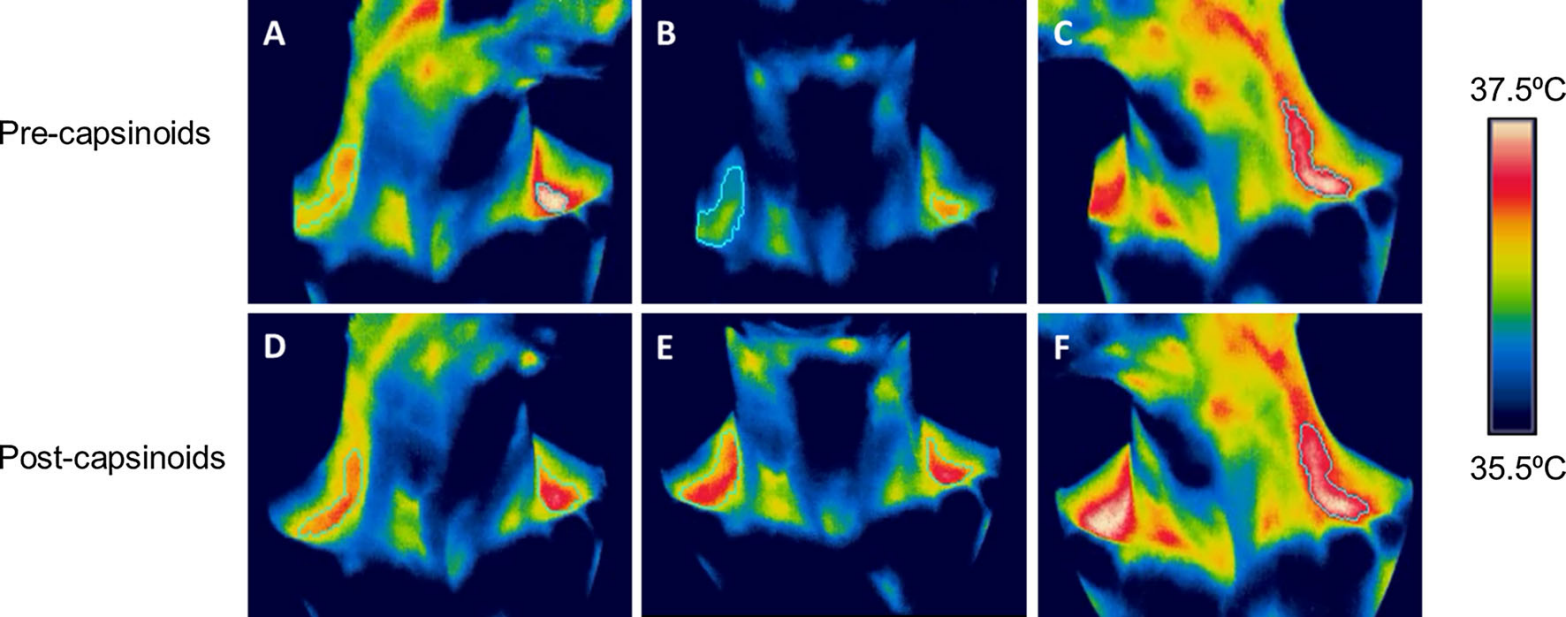
Thermal images pre- and post-ingestion of capsinoids. Representative images taken at baseline (**a**, **b**, **c**) and at 2.5 h (**d**, **e**, **f**) following ingestion of capsinoids, showing increase in the area and temperature of the thermally active region. The subject’s head was turned to his left in **a** and **d**, was facing forward in **b** and **e**, and was turned to his right in **c** and **f**.* Green contour lines* circumscribe the ROIs over the C-SCV regions, *white pixels* >37.4 °C, *red pixels* 36.8–37.4 °C, *yellow pixels* 36.5–36.8 °C. Color figure online

Figure 7a, b shows the change in C-SCV heat output in the low-BAT and high-BAT groups during the 2.5-h period following the ingestion of capsinoids, and Fig.  8 shows the effect of consuming capsinoids on C-SCV heat production based on the results of our IRT protocol. Similar to the results for EE and fat oxidation, the gSEM analysis revealed that the high-BAT group had a significantly higher average heat output after adjusting for baseline values during the 2.5-h period following the ingestion of capsinoids than following the ingestion of placebo (coefficient 0.48; *p* = 0.01); however, once again, the low-BAT group showed no difference in heat output between the consumption of capsinoids and placebo (coefficient 0.09; *p* = 51) (ESM Supplementary Table 3).

### C-SCV heat production measured by IRT correlated with expected changes in metabolic parameters

If IRT quantifies heat production over C-SCV regions and is a potential indicator of BAT activation, the heat output measured would be expected to correlate with the metabolic consequences of active BAT. Given that glucose uptake, lipolysis, fat oxidation, and uncoupling of oxidative phosphorylation with resultant EE in the form heat dissipation occurs in stimulated BAT, it is not surprising that the presence of BAT correlates inversely to fasting glycemia and directly to metabolic rate [33–35]. In our study, we observed a significant inverse correlation between subjects’ mean fasting blood glucose level and heat output in the * Green contour lines* circumscribe the ROIs over the C-SCV regions in response to cold (*r* = −0.51, *p* = 0.011) (see ESM Fig. S1). We also observed a significant correlation between the degree of heat production in the C-SCV region and BMR (*r* = +0.44, *p* = 0.017) (see ESM Fig. S2). Moreover, most skin surfaces overlying non-BAT regions and highly vascularized skin in the peripheries (e.g.. ear lobe) tend to undergo vasoconstriction during cold exposure as part of the defense against hypothermia. Increases in surface skin temperature in response to cold stimulation is thus unexpected, except where this is associated with BAT activation which in turn is coupled to increased perfusion to match its increased metabolic demands (ESM Fig. S3a, b). Together, these findings support our IRT methodology for the assessment of BAT activation.

## Discussion

Here we report our use of a new method that employs IRT to quantify changes in heat production in the C-SCV region as a reflection of supraclavicular BAT activity. This new method differs in important ways from IRT methods used in previous studies to measure supraclavicular skin temperature as a reflection of brown fat thermogenesis [21–24]. For example, in contrast to previous studies, we imaged the anterolateral C-SCV areas on both sides of the neck (Fig. 3). This imaging protocol allowed us to image a larger area of skin overlaying the BAT depots in the C-SCV regions that would otherwise be obscured if subjects faced the camera directly. We observed a distinct area of skin with a higher temperature than that in neighboring skin, which extended from the SCV region along the lateral neck. This observation is consistent with the macroscopic anatomical description of cervical BAT in humans [36, 37].

We also developed a new algorithm to quantify changes in IRT based on a SRG image processing technique. Our segmentation algorithm identified circumscribed enlarging ROIs characterized by a dynamically escalating and higher mean temperature in comparison to neighboring skin which occurred in response to cold stimulation and to the ingestion of capsinoids. As the segmentation contour line does not represent isotherms but is based on a sharp change in the dermal temperature gradient, the ROI boundary demarcates the approximate size of the underlying BAT depot. By taking into account changes in both temperature and area, our method better reflects heat energy output by BAT rather than merely measuring skin temperatures. Another difference in our protocol from those used in previously published studies was the use of IR video sequences rather than single-frame IR images; to this end, we used 5 s of video data, comprising a total of 150 frames, for each analysis, such that the temperature and area values for the ROIs can be averaged out across all the frames to measure IR energy flux with greater accuracy compared to single-frame analysis.

Recent studies using either IRT or iButtons, a temperature logging advice, have shown that supraclavicular skin temperatures significantly increase upon cold exposure [20, 22, 38]. The aim of our study was therefore to validate our new IRT method for the detection of heat production in the C-SCV region in the context of cold stimulation as an environmental intervention. Subjects were exposed to a local cooling protocol, namely, placing hands and feet in cold water, and heat production in the C-SCV region at the start and end of the 5-min cooling protocol was calculated to determine the change in heat production in the C-SCV region induced by cold stimulation. Taking into consideration an estimated prevalence of active BAT in humans, determined in previous PET studies, of between 30 and 100 % [39] and the broad variation in cold-induced heat energy output observed in our subject population, we divided the subjects into low-BAT (*n* = 18) and high-BAT (*n* = 6) groups, such that subjects in the high-BAT group displayed a significantly greater percentage increase in heat output in response to cold compared to the low-BAT group (Fig. 4). Both subject groups had similar anthropometric measurements (Table 1), thus allowing a comparison of EE and skin temperature responses without having to take into consideration any possible effects of adiposity on the responses.

In addition to studying the effect of cold stimulation, we sought to validate our method of BAT detection in a context of a dietary intervention to assess its feasibility as a new method for the discovery of food and nutraceuticals with BAT-stimulatory properties. A previous study using ^18^F-FDG PET reported that the ingestion of capsinoids increases EE in adult humans through the activation of BAT, based on evidence that the thermogenic effect of capsinoids was only observed in the BAT-positive group but not the BAT-negative group [17]. Our results show that the ingestion of capsinoids elicited a significant increase in EE compared to the ingestion of placebo in the high-BAT group, but no difference in EE was detected between the ingestion of capsinoids and placebo in the low-BAT group (Fig. 5, ESM Table 1). In both groups, placebo ingestion resulted in a small rise in EE, which likely reflects the contribution of the thermic effect of food (an obligatory exothermic chemical process via the digestive breakdown of macromolecular bonds) [40] due to the consumption of white rice that was provided together with the capsinoids in our study to mimic its consumption as part of a traditional meal. Similar to EE, fat oxidation was significantly higher after the consumption of capsinoids compared to the consumption of placebo only in the high-BAT group, whereas no significant difference was detected for the low-BAT group (Fig. 6, ESM Table 2). Our results are therefore consistent with the previous observation that capsinoid-induced thermogenesis depends on BAT activation.

We then examined whether our IRT method could detect BAT activation induced by capsinoids. Similar to our results for EE and fat oxidation, those for the effect of capsinoids show that the ingestion of capsinoids elicited a significant increase in heat production in the C-SCV region compared to the ingestion of placebo only in the high-BAT group, but not in the low-BAT group (Fig. 7, ESM Table 3). Our findings that capsinoids induced significant increases in EE, fat oxidation, and heat production in the C-SCV regions in the high-BAT group but not in the low-BAT group are consistent with the hypothesis that capsinoids stimulate thermogenesis through BAT activation [17]. As such, these results validate our IRT method for detecting BAT activity since our high-BAT group likely represents individuals who are BAT-positive and thus responsive to the thermogenic effects of capsinoids while the low-BAT group represents individuals lacking activatable BAT and thus unresponsive to capsinoids. Furthermore, it is remarkable that the findings in the high-BAT group were statistically significant despite its much smaller sample size compared to the low-BAT group, implying that the effect sizes of the high-BAT group were large and that the biological events triggered by capsinoids most probably occurred as a result of actual BAT activation.

Given that the inverse link between quantity of functional BAT and glucose homeostasis has been established [41], our replication of this finding (ESM Fig. S1) supports our method as a reliable BAT imaging tool. The correlation of the degree of heat output in the C-SCV region to changes in BMR (ESM Fig. S2) lends further credence to the utility of both our novel IRT method as well as indirect calorimetry as BAT activity assessment tools. The results of this study provide the rationale to propose IRT as a feasible method for detecting BAT activation, thereby providing a safer and cheaper alternative to PET/CT. Since PET/CT-based BAT detection is often performed after a 2-h cooling protocol and is restricted to a single time domain of capture depending on the kinetics of the radiopharmaceutical, IRT offers clear advantages for BAT assessment by its ability to serially measure real-time changes in BAT responses of an individual to a modest cold stimulus or food intake. In addition, the equipment and study costs are significantly less than those of PET/CT imaging and IRT facilities can be located within a clinical research unit. Most importantly, IRT can be performed numerous times, including serially, on individual subjects since it does not involve ionizing radiation exposure, as does PET/CT.

There are a number of limitations to the present study. Our findings could be strengthened by validation against FDG-PET/CT in order to show that the heat production in the C-SCV region as measured by our IRT method correlates with measurements of BAT activity by the current reference method. It should be noted that IRT likely underestimates the heat energy output from BAT since it detects changes in temperature at the skin surface, which is a result of heat transfer from the underlying BAT depots along a temperature gradient, and could be confounded by subcutaneous adipose tissue thickness [42]. One common criticism of IRT is that the increase in heat emission observed over the C-SCV regions is purely due to increased blood flow rather than due to BAT activation. While we acknowledge that increased blood flow can account for an increase in heat emission detected by IRT, at least two facts support that the increased IR heat power over the C-SCV is related to BAT activation. First, it should be noted that BAT activation is definitely accompanied by increased blood flow due to the oxygen and nutrient demands of thermogenesis [43]. In other words, increased BAT thermogenesis is by necessity coupled to increased perfusion. However, it must be noted that during cold exposure, the normal and expected physiological thermoregulatory response to conserve body heat is to shunt blood flow away from the skin to minimize further heat loss from the skin surface [44]. Indeed, the skin over the body surface generally reacts to cold exposure by vasoconstriction which reduces IR heat loss. This implies that any increase in local blood flow over the skin during cold exposure is counterintuitive and paradoxical and that any increase in skin temperature during cold exposure is thus biologically maladaptive and untenable unless that skin region possesses thermogenic tissues, such as activatable BAT underneath it.

Future work will involve further development and optimization of IRT image processing tools for BAT quantification. These data-rich IR video sequences (~1.4 GB for a 5-min sequence) require high computing power and the development of processing algorithms to obtain meaningful results. One of the challenges that the present study faced was achieving proper segmentation results for detecting the C-SCV BAT of human subjects. The threshold parameter for the segmentation algorithm was manually optimized for individual subjects to achieve reliable segmentation, since any single pre-set threshold for the algorithm failed to generate reliable segmentations across all subjects. Future work will involve fully automated computer algorithms to eliminate user dependence and thus allow for greater reproducibility.

In conclusion, this study provides evidence supporting the use of a newly developed method of quantitative IRT for studying BAT function in humans which can be used in a serial fashion to assess BAT activation over multiple time points in individual subjects. Due to the low cost, safety, and the ability to use IRT within clinical research laboratories, this new method will be useful for screening an array of foods, drugs, and environmental stimuli for their ability to activate BAT, as well as to quantify BAT activity as a way to determine the relative ability of different stimuli to activate BAT.

## Electronic supplementary material

Below is the link to the electronic supplementary material.

Supplementary material 1 (DOC 863 kb)

Supplementary material 2 (DOCX 16 kb)
