# Supplementary material for: A new method of infrared thermography for quantification of brown adipose tissue activation in healthy adults (TACTICAL): a randomized trial
Source: J Physiol Sci. 2016 Jul 21;67(3):395–406. doi: 10.1007/s12576-016-0472-1 (PMC5477687; doi:10.1007/s12576-016-0472-1)
Supplement: Supplementary file 1 — Supplementary material 1 (DOC 863 kb) [file 12576_2016_472_MOESM1_ESM.doc]

# SUPPLEMENTARY FIGURES

**
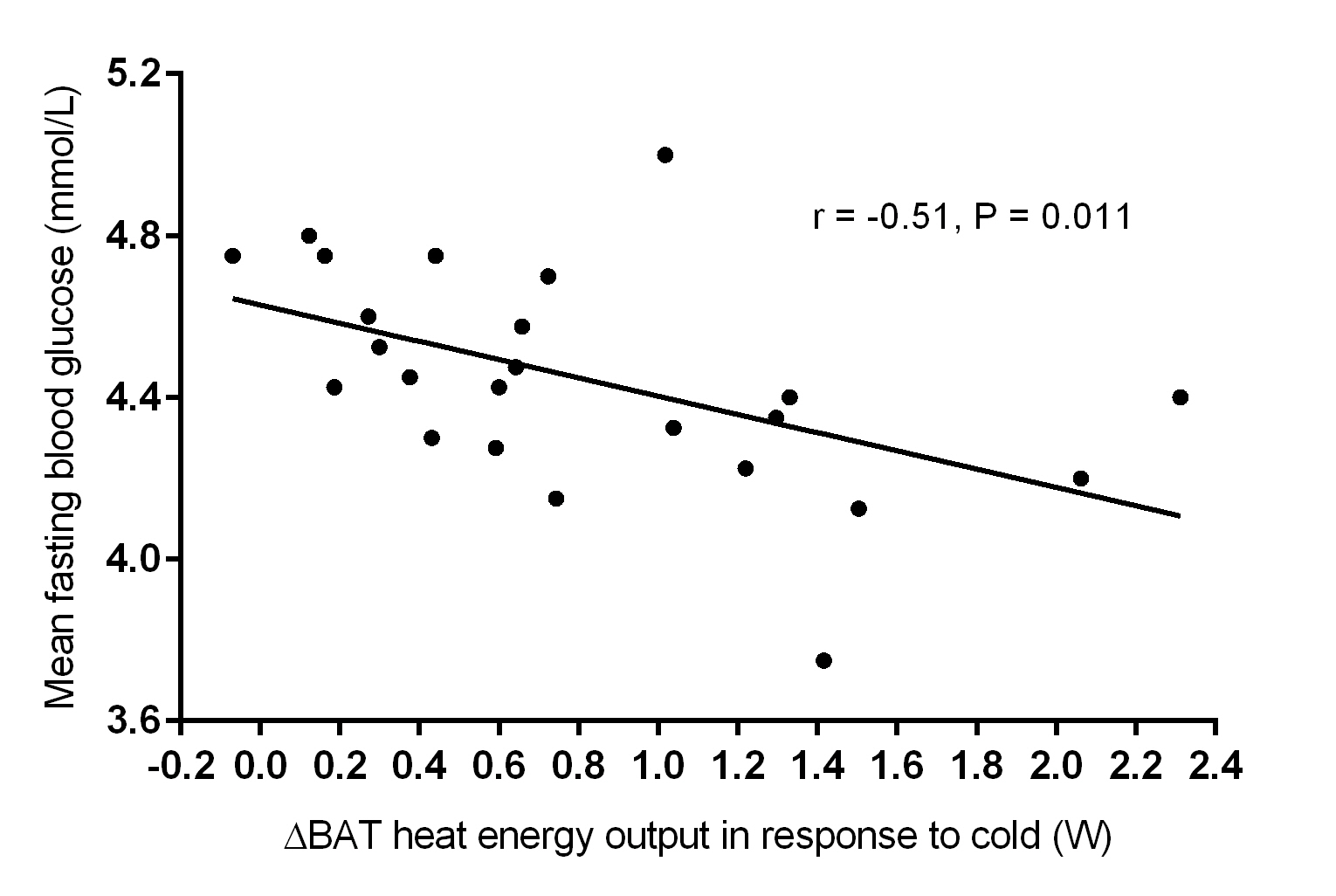
**

**Supplementary Fig. S1** Subject’s mean fasting blood glucose is inversely correlated with the change in C-SCV heat energy output during cold challenge (r = -0.51, P = 0.011).

**
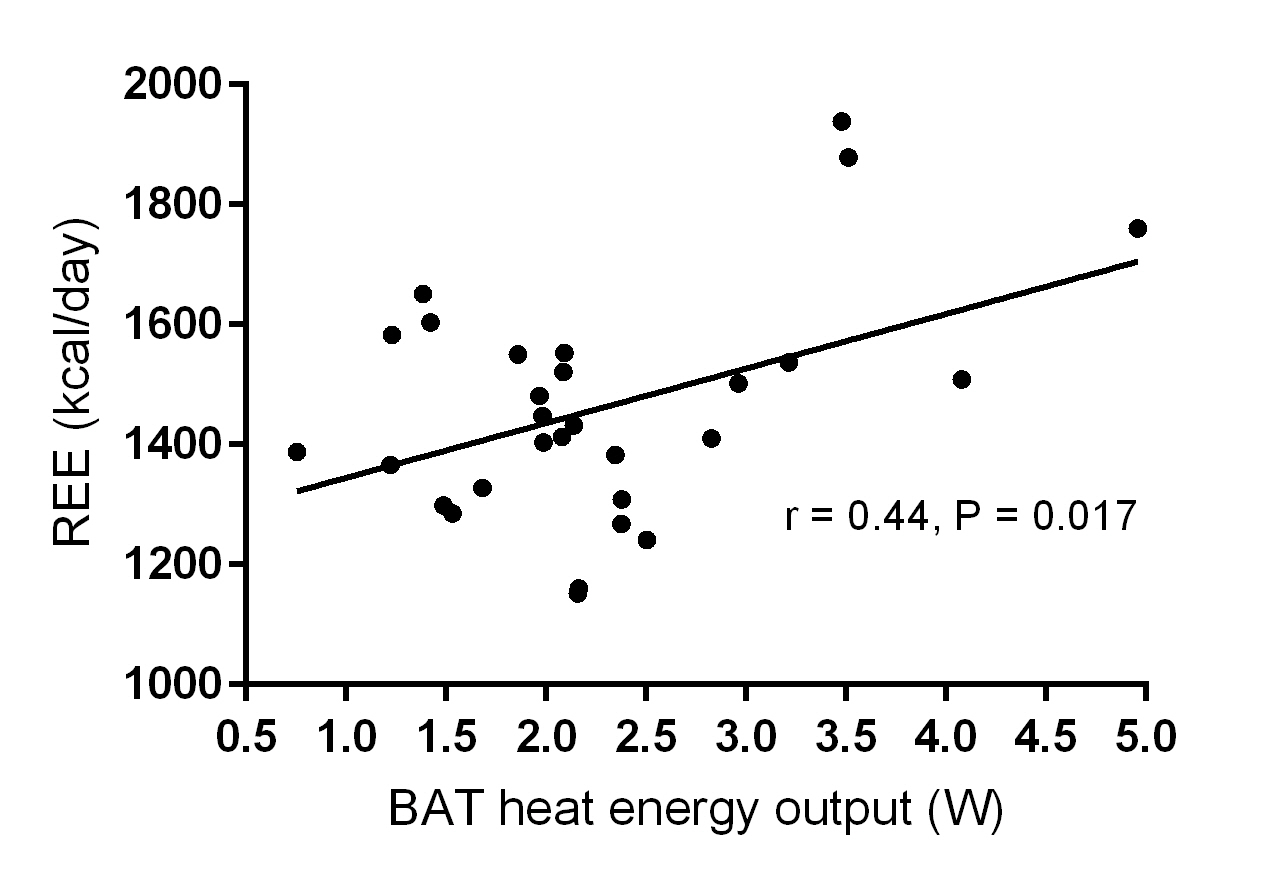
**

**Supplementary Fig. S2** Positive correlation between resting energy expenditure (REE) and C-SCV heat energy output (r = +0.44, P = 0.017).

**
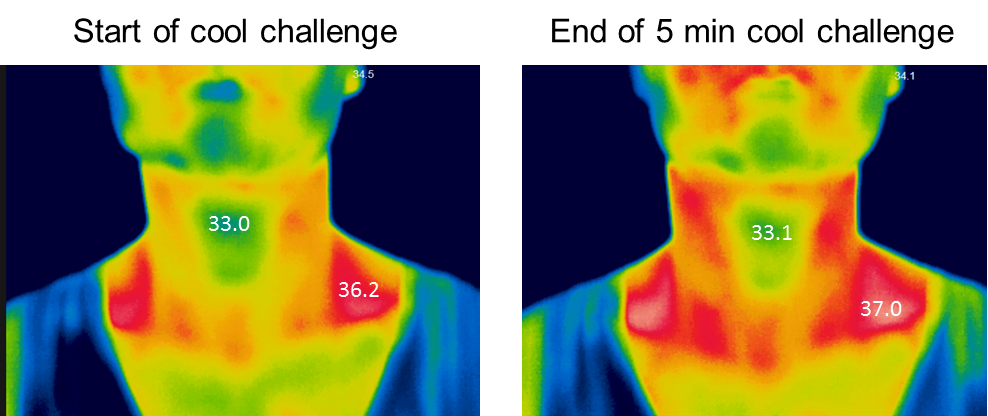
**

S3a

S3b

**Supplementary Fig. S3a and S3b.** Temperature responses to cold exposure vary as a function of skin vascularity and BAT absence/presence. Prior to cold stimulation, the left ear lobe (capillary-rich skin) showed a baseline temperature of 34.5º C while the skin overlying the trachea (less densely vascularized) was 33.0º C and the supraclavicular skin overlying BAT was 36.2ºC. Towards the end of the 5 min cold challenge, the same left ear lobe had cooled down to 34.1º C through adaptive thermoregulatory vasoconstriction to shunt peripheral blood flow away from the surface to reduce heat loss. The skin of a less vascularized non-BAT region over the trachea was minimally perturbed whereas the skin overlying known BAT sites rose markedly to 37.0º C in stark contrast to highly vascularized non-BAT skin peripheries which generally become cooler from vasoconstriction as a defense against decline in core temperature.
