# Supplementary material for: A new method of infrared thermography for quantification of brown adipose tissue activation in healthy adults (TACTICAL): a randomized trial
Source: J Physiol Sci. 2016 Jul 21;67(3):395–406. doi: 10.1007/s12576-016-0472-1 (PMC5477687; doi:10.1007/s12576-016-0472-1)
Supplement: Supplementary file 2 — Supplementary material 2 (DOCX 16 kb) [file 12576_2016_472_MOESM2_ESM.docx]

# SUPPLEMENTARY TABLES

**Supplementary Table 1. Statistical (gSEM) analysis of energy expenditure during capsinoids trial**

|  | **All**  Coefficient (95% C.I.) | **Low-BAT**  Coefficient (95% C.I.) | **High-BAT**  Coefficient (95% C.I.) |
| --- | --- | --- | --- |
| Baseline | 0.97 (0.84 — 1.10) * | 0.96 (0.86 — 1.06) * | 1.00 (0.65 — 1.34) * |
| Time (hour)  0.5  1.0  1.5  2.0  2.5 | Reference  18.60 (-4.03 — 41.23)  9.24 (-15.35 — 33.82)  -10.13 (-33.59 — 13.33)  -26.55 (-51.90 — -1.21) * | Reference  9.31 (-17.77 — 36.40)  14.18 (-17.65 — 46.00)  -12.18 (-41.32 — 16.97)  -23.64 (-57.00 — 9.72) | Reference  44.60 (9.89 — 79.31) *  -4.75 (-30.55 — 21.04)  -4.40 (-44.89 — 36.09)  -34.70 (-62.51 — -6.89) * |
| Treatment  Placebo  Capsinoids | Reference  37.09 (3.90 — 70.29) * | Reference  26.30 (-13.34 — 65.94) | Reference  66.90 (16.21 — 117.59) * |

*Significant at 5%.

**Supplementary Table 2. Statistical (gSEM) analysis of fat oxidation during capsinoids trial**

|  | **All**  Coefficient (95% C.I.) | **Low-BAT**  Coefficient (95% C.I.) | **High-BAT**  Coefficient (95% C.I.) |
| --- | --- | --- | --- |
| Baseline | 0.91 (0.78 — 1.05) * | 0.94 (0.73 — 1.15) * | 0.88 (0.73 — 1.04) * |
| Time (hour)  0.5  1.0  1.5  2.0  2.5 | Reference  -13.51 (-17.22 — -9.80) *  -13.18 (-17.61 — -8.76) *  -9.66 (-14.34 — -4.99) *  -8.68 (-13.19 — -4.17) * | Reference  -14.29 (-18.88 — -9.70) *  -15.33 (-20.61 — -10.05) *  -11.17 (-16.77 — -5.58) *  -9.16 (-14.70 — -3.62) * | Reference  -11.49 (-17.89 — -5.08) *  -7.60 (-13.99 — -1.21) *  -5.74 (-14.11 — 2.62)  -7.43 (-15.81 — 0.95) * |
| Treatment  Placebo  Capsinoids | Reference  7.06 (-0.27 — 14.39) | Reference  5.17 (-4.18 — 14.52) | Reference  11.88 (1.15 — 22.60) * |

*Significant at 5%.

**Supplementary Table 3: Statistical (gSEM) analysis of C-SCV heat production during capsinoids trial**

|  | **All**  Coefficient (95% C.I.) | **Low-BAT**  Coefficient (95% C.I.) | **High-BAT**  Coefficient (95% C.I.) |
| --- | --- | --- | --- |
| Baseline | 1.12 (1.07 — 1.17) * | 1.10 (1.03 — 1.16) * | 1.26 (1.24 — 1.29) * |
| Time (hour)  0.5  1.0  1.5  2.0  2.5 | Reference  0.10 (-0.09 — 0.29)  0.22 (-0.01 — 0.45)  0.28 0.08 — 0.49) *  0.35 (0.06 — 0.64) * | Reference  0.11 (-0.12 — 0.35)  0.09 (-0.12 — 0.30)  0.22 (-0.00 — 0.44)  0.33 (0.04 — 0.63) * | Reference  0.07 (-0.29 — 0.43)  0.60 (0.04 — 1.17) *  0.51 (0.05 — 0.97) *  0.39 (-0.41 — 1.19) |
| Treatment  Placebo  Capsinoids | Reference  0.17 (-0.05 — 0.38) | Reference  0.09 (-0.17 — 0.35) | Reference  0.48 (0.10 — 0.86) * |

*Significant at 5%.
